# Complete genome sequence of *Staphylococcus casei* strain DSM 15096

**DOI:** 10.1099/acmi.0.000656.v2

**Published:** 2023-07-26

**Authors:** Caitlin Wildsmith, Jonathan C. Thomas, David Negus

**Affiliations:** ^1^​ Department of Biosciences, Nottingham Trent University, Nottingham, UK

**Keywords:** CoNS, hybrid assembly, illumina, nanopore

## Abstract

We present the first complete genome sequence of the species *Staphylococcus casei *. Strain DSM 15096 was sequenced with a hybrid approach using Oxford Nanopore Technologies long-read sequencing and Illumina short-read sequencing. The assembled sequences produced a 2 808 898 bp chromosomal molecule containing 2705 predicted genes, plus eight plasmids.

## Data Summary

All data are available under BioProject accession number PRJNA971866 and BioSample accession number SAMN35056232. The genome and plasmids were deposited under GenBank accession numbers CP125718-CP125726.Nanopore basecalled data and Illumina raw read data were submitted to SRA and are available via accession numbers SRR24632484 and SRR24632485 respectively.

## Announcement

*Staphylococcus casei* is a coagulase-negative staphylococcal species that was originally isolated from surface-ripened cheese in 1997 and classified as *Staphylococcus succinus* subsp. *casei* in 2002 by Place *et al.* [1]. Later phylogenomic analysis of the *Staphylococcaceae* family by Madhaiyan *et al.* determined that *S. succinus* subsp. *casei* should be reassigned as a novel species, *S. casei* [2]. This new nomenclatural status was approved by the International Code of Nomenclature of Prokaryotes [3]. The type strain of the species is *S. casei* DSM 15096 (SB72^T^) and its original genome sequence was deposited with NCBI in 2018 under Genbank assembly accession GCA_002902445.1 [4]. The genome sequence has however been flagged by NCBI as misassembled and the assembly was excluded from RefSeq. There are currently no other genome sequences publicly available for either *S. succinus* subsp. *casei* or *S. casei *. To address the lack of an available, high-quality genome sequence for this species, we purchased *S. casei* DSM 15096 as a freeze-dried sample from the German Collection of Microorganisms and cell cultures (https://www.dsmz.de/) and applied a hybrid assembly approach using Oxford Nanopore Technologies long-read sequencing and Illumina short-read sequencing to produce a complete genome sequence.

The strain was revived in Tryptone soy broth (TSB; Oxoid) and streaked onto Tryptone soy agar (Oxoid) for single colonies. A single colony was transferred to 5 ml of TSB and incubated overnight at 37 °C with orbital shaking. The liquid culture (1 ml) was centrifuged, and the cell pellet was resuspended in 200 µl of enzymatic lysis buffer (20 mM Tris-Cl, pH 8.0, 2 mM sodium EDTA, 1.2 % Triton X-100, 20 mg ml^−1^ lysozyme, 25 µg ml^−1^ lysostaphin) and incubated at 37 °C for 1 h. DNA was extracted using a QIAGEN DNeasy blood and tissue kit (QIAGEN) following the Gram-positive extraction protocol. Illumina short-read sequencing was conducted by MicrobesNG (https://microbesng.com/). The genome was sequenced using the Illumina HiSeq 2500 platform and 2×250 bp paired-end technology producing 551534 paired-end reads. Nanopore library preparation was performed using the ligation sequencing kit SQK-LSK109 (Oxford Nanopore Technologies) and native barcoding and sequenced on a MinION flow cell (R.9.4.1).

Sequencing reads were assembled using default parameters for all software except where otherwise specified. Raw nanopore sequencing reads were basecalled using the super high accuracy (SUP) model of Guppy v6.4.2. Adapter sequences were trimmed using Porechop v0.2.4 [5] with middle and end thresholds of 85 and 95 %, respectively. Reads were filtered based on length (>1 Kb) using Filtlong v0.2 [6]. Reads were assembled using Flye v.2.9.1 [7] and polished with four iterations of Racon v1.5.0 [8] (with parameters -m 2 -g -2 -u) followed by Medaka v1.7.2 [9] and Homopolish v0.3.4 [10].

Adapter sequences were removed from raw Illumina reads using Trimmomatic v0.39 [11]. Illumina sequence reads were trimmed based on quality with Sickle v1.33 [12]. Reads were trimmed where the mean Phred score dropped below Q15 using a sliding-window approach [12]. The nanopore assembly was error corrected with trimmed Illumina reads using Polypolish v0.5.0 [13], POLCA (part of MaSuRCA package v4.0.9) [14] and NextPolish v1.4.1 [15]. The genome was manually trimmed and reorientated to start at *dnaA*.

The complete bacterial genome was annotated using the NCBI prokaryotic genome annotation pipeline version 6.5. The chromosome was predicted to encode 2705 genes, 25 rRNAs, 61 tRNAs and 27 pseudogenes. The strain contained eight plasmids that ranged in sizes from 32 242 bp to 2 505 bp. Assembly quality was determined with CheckM v1.1.3 [16] with a completeness of 99.45 %, contamination of 1.38%, an N50 of 2 808 898 bp and G+C content of 33.23 %. Average nucleotide identity (ANI) against the existing misassembled *S. casei* type strain genome (GCA_002902445.1) showed 99.89 % similarity and was calculated using FastANI v1.1 [17]. ANI between the type strain of the species *S. succinus* DSM 14614 (AMG-D1^T^) and the chromosome of our *S. casei* assembly showed 95.58 % similarity. This supports the finding of Madhaiyan *et al*. who reported 95.6 % ANI between the existing *S. casei* type strain genome and *S. succinus* DSM 14614 (AMG-D1^T^).
